# Experiences of nursing students who are victims of dating violence: a qualitative study

**DOI:** 10.1186/s12912-023-01688-w

**Published:** 2024-01-09

**Authors:** Esperanza Barroso-Corroto, José Alberto Laredo-Aguilera, Ana Isabel Cobo-Cuenca, Juan Manuel Carmona-Torres

**Affiliations:** 1 Hospital Universitario de Toledo, Toledo, 45007 Spain; 2 Grupo de Investigación Multidisciplinar en Cuidados, Universidad de Castilla-La Mancha, Toledo, 45071 Spain; 3 Facultad de Fisioterapia y Enfermería, Universidad de Castilla-La Mancha, Toledo, 45071 Spain

**Keywords:** Dating violence, Intimate Partner Violence, Nursing students, Victims, College students, Anxiety, Qualitative

## Abstract

**Background:**

Dating Violence (DV) is a type of Intimate Partner Violence that occurs between young people, and they are those behaviours that cause physical, sexual or psychological harm.

**Objective/aim:**

To know the experience of university students around dating violence.

**Design and methods:**

Qualitative study with a phenomenological approach was conducted through semi-structured individual interviews with nursing students’ victims of dating violence with the same starting categories. The public involve in this study were nursing students who freely agreed to participate in the interviews and gave their informed consent.

**Results:**

Eleven nursing students participated, the sample was heterogeneous for gender and sexual diversity. Obtaining results about their experience with dating violence, manifestations of dating violence and cyber violence in their relationships, consequences, formal and informal help seeking and proposals for help as nursing students, among others.

**Conclusion:**

Dating violence is a serious problem that seriously affects the victims and requires the creation of prevention programs. The experiences of university students about DV are mainly painful experiences, with serious consequences for those involved, needing help from their close environment and professional help to overcome the problems generated by their partners.

**Implications:**

It is important due to the high prevalence of this phenomenon, also among nursing students, to provide key points to future health professionals and victims of dating violence on the correct way to act against violence due to lack of knowledge on the subject. This study clarifies the experiences of dating violence and how to offer help to victims from the informal and professional sphere.

**Trial registration:**

This study was approved by the Ethics Committee of Clinical Research of the Health Area of Talavera de la Reina (Toledo) with code 01/2021.

**Supplementary Information:**

The online version contains supplementary material available at 10.1186/s12912-023-01688-w.

## Background

Dating violence (DV), or intimate partner violence as referred to by the World Health Organization (WHO), is a type of abuse that tends to occur among young people [1] and is understood as behaviors within an intimate relationship that cause physical, sexual or psychological harm, including acts of physical aggression, sexual coercion, psychological abuse and control behaviors [2, 3]. Importantly, the term refers to a broad array of relationships, from a highly committed relationship to a relationship between simple acquaintances [4].

DV can occur through electronic media and the internet [5]. Electronic DV involves actions of control, harassment, abuse, coercion, threats, and/or humiliation of a partner through digital devices [6–8].

Structural violence refers to the violence that is intrinsic to the social, political, and economic structures of a society. There is a relationship between structural violence and dating violence, as the former contributes to the normalization of violence in dating and the perpetuation of gender inequalities[9].

Furthermore, control and coercion are transmitted structurally between peers, thus preserving psychological violence through social norms[10].

According to a systematic review on the prevalence of DV among adolescents in Europe since 2010 [11], there are no significant differences by gender, and the prevalence rates among different countries are highly variable, both in terms of being a perpetrator and a victim of violence. For example, the review indicates that between 25.1% and 95.5% of women and between 19.0% and 94.5% of men are victims of psychological violence, between 4.7% and 32.9% of women and between 7.3% and 29.8% of men are victims of physical violence, and between 7.8% and 41% of women and between 6.1% and 31.0% of men are victims of sexual violence. In addition, similar patterns are seen for the types of DV perpetrated. These highly variable results are similar to those obtained in other studies carried out in other countries, where prevalence ranges from 27.7% to 70.7%, although many of the studies do not specify the type of violence or gender [12]. Additionally, agreeing with studies carried out in Spain [13, 14], we also found high prevalence rates among young adults between 18 and 24 years of age.

Being a victim of DV carries negative consequences for those who suffer it. These include higher levels of depression, anxiety, suicidal thoughts and poor academic outcomes [15].

Among the risk factors that have been associated with experiencing DV [16–19] are the consumption of substances, especially alcohol and cannabis; marked personality traits, feelings of anger, anxiety or sadness; antisocial behaviors; noncommitted casual relationships (greater tendency to commit sexual violence); risky sexual behaviors, for example, early sexual relations, pornography or high number of sexual partners (the more partners, the greater the risk is of suffering or perpetrating violence); and having been a victim or having witnessed any other type of violence. Other risk factors have also been identified: traditional ideas of gender roles, seeking an ideal of romantic love and having sexist behaviors [20].

There are also protective factors for DV, among which are high levels of empathy, having high academic grades, verbal intelligence, disagreeing with any type of violent behavior, being engaged in studies and having a good relationship with family [21].

The pathway to help for people suffering from DV starts with defining the problem and deciding to seek help, whether formal or informal. A social support network to turn to as informal help is critical and is generally made up of close friends and relatives; this informal help can then lead to seeking formal help when needed [22]. Talking about DV is usually the first step in seeking social support for DV victims, with informal support being more common [23].

DV is a current problem, with a high incidence in all the quantitative studies found and with serious consequences for those who are victims of this issue. However, few studies focus on collecting experiences, understanding what victims feel, and determining how people who are victims of DV react.

Among the previous qualitative studies, to our knowledge, there is no known study that focuses on how the victims of DV live, what leads them to not be able to identify whether they are suffering violence, what decisions they make and why they make these decisions about their relationships. We did find studies on gender differences in experiencing DV [24, 25], on the help-seeking process, and the peer-help process [22, 26–29]. Therefore, it is of special interest to investigate the experiences of DV among young university students to help establish prevention plans and solve this global problem.

For this reason, the main objective of this study is to determine the experience of university students with regard to DV.

## Methodology

### Design

This study was conducted using a qualitative methodology with a qualitative phenomenological approach, allowing a deeper analysis of the details and of how the people involved in DV convey their experiences, associated factors, consequences, and forms of help, so as to improve the comprehension and understanding of this issue.

The phenomenological study was carried out through semistructured individual in-depth interviews with people of both sexes. This study is a continuation of a previous study [13] in which the prevalence of DV in college students was known. Participants who had suffered and/or witnessed DV directly were invited to participate in the in-depth interviews. The interviews lasted approximately one hour per participant.

The semistructured individual interview methodology was chosen because it allows interviews to focus specifically on DV and provides the researcher with autonomy to ask and explore relevant factors of interest while the interview is taking place [30].

The interview used was developed for this study. The Interview guide has been provided as supplementary file.

This study is part of a research project on DV among university students that included a quantitative pilot study that evaluated the prevalence of DV in a Spanish University and the associated variables in nursing students [13].

The interviews were carried out by Barroso-Corroto E. PhD student who has received training in qualitative methodology before starting the study. She is doing her thesis on dating violence in college students. She is interested in implement a training program to prevent intimate partner violence.

The rest of the research group are professors with PhDs in Nursing at the University of Castilla-La Manchawith extensive research experience.

The scientific writing of this research was carried out taking into account the objectives of the COREQ guide [31].

### Participants and recruitment

Participants were selected by convenience and voluntary sampling using information obtained through a Google Forms questionnaire that measured the prevalence of DV and other associated variables at the university, allowing the participants to be divided into victims and perpetrators of DV; at the end of the first section of the questionnaire, individuals agreed to participate in the qualitative study phase. Of the 248 participants, 132 were victims of DV, and only 20 agreed to participate in the interviews, with 11 who actually participated. The others did not respond to the emails sent out to schedule the interviews or did not show up once scheduled.

The number of interviews was estimated taking as reference the results obtained by an study conducted in Spain [32] in which only 5 of 277 victims of intimate partner abuse were willing for the in-depth interview. Based on this, at least 10 interviews were estimated as necessary.

The inclusion criteria were be a nursing student in the 2021/2022 academic year at the university, having been a victim of DV or have had close experiences with victims of DV. In addition to having participated in the previous study.

There was not any relationship between the interviewer and the participants before the study. The participants knew the identity of the interviewer and all questions about the interviewer related to the study where solved when asked.

### Data analysis and study procedures

The interviews were carried out online through the Microsoft Teams platform between February and June 2022. Verbal informed consent to record the interviews was requested from all participants. There was no presence of non-participants during the interviews. No repeated interviews were carried out to any participant. No field notes were made during the interviews since they were being recorded. The interviews lasted approximately 1 h per participant.

Tests were carried out with the application of Microsoft Teams among the researchers to check the options for using this tool.A pilot interview were carried to test the starting categories and it was not taken into account for the data analysis.

The starting topics were the same for all students. They were asked about their experience with DV, whether they could identify any violent behaviors in their relationship, whether they had observed or suffered violent behaviors through social networks, their experiences in seeking help, coping with the situation, consequences and opinions, among other topics. Data saturation was achieved for most of the baseline categories. Lack of participation was a problem in reaching full data saturation.

Once the interviews were finished, the transcriptions were shown to all participants at the end of each interview to do any comments or corrections needed. The software ATLAS.Ti version 7 [33] was used for the synthesis and qualitative analysis of the transcripts obtained.

A thematic synthesis of the data from the in-depth interviews was carried out. The data analysis process was conducted based on the steps proposed by Graneheim and Lundman [34]. The in-depth interviews with the participants were recorded. A verbatim transcription of all audio recordings was made. To ensure confidentiality, data protection and anonymity of the participants, all files and recordings were stored on the interviewer's PC, protecting the file with a password. Only the interviewer had access to the recordings and transcripts. Copies of the recordings were deleted once all anonymous transcripts were completed.

The researchers transcribed the interview word for word and conducted a data analysis after conducting the interview. Then, the total text was read several times to get a general idea of the content of the interview. The total text was considered as the unit of analysis and shorter parts such as phrases, sentences or paragraphs, which had a concept related to the starting categories, were considered as the unit of meaning. Each unit of meaning was coded. The codes were classified into subcategories according to their similarities and differences. For this purpose, ATLAS.ti version 7 software was used. A content analysis was carried out through the categorization and coding of verbal data, a total of 46 different codes were used and grouped according to the theme: types of violence (Physical, psychological, social, and ciberg dating violence), consequences of suffering DV, justification/normalization of DV and help seeking. Other codes used were search for solutions, feelings, breakup of the relationship, manipulation, becoming aware of the situation, control, blackmail, romantic love. At the same time, an inductive analysis of the narrative content of the interviews was carried out.

## Results

A total of 11 nursing students participated, of whom four were men and seven were women. Regarding sexual orientation, one participant was bisexual, one participant was homosexual, and nine participants were heterosexual. The participants were between 19 and 25 years old. All the participants belonged to the Spanish culture, raised with parents of Spanish nationality and all spoke Spanish as their mother tongue. Among the participants, four indicated they had not suffered DV but had closely related experiences, and seven were victims of DV. Among the victims, one was not aware of having suffered violence despite her positive score in the violence questionnaire carried out in the previous phase of this study [13].

When asked about their experience in relation to DV, most of the victims began by stating that they had not suffered physical violence but had experienced psychological and verbal abuse.

"*For me, it was my ex-partner, and there was no violence, no physical violence, but there was psychological abuse, a lot… That is, to the point that in the end, I had an emotional dependence on the other person, and feeling it all the time, that was the worst. Blamed for everything and told that there was something wrong with me. And I couldn’t leave because, of course, I felt that if I lost him… It was like nobody else in the world was going to love me because I was so horrible. Or that’s what that person made me feel. And, nothing. Let me remember, so much disrespect, insults, evil looks… never-ending*.” Participant 3. Heterosexual woman, victim of DV.

*“Let’s see, the relationship I had was with a woman, in fact. So, let’s see, my experience with violence, if it can be called violence, has been more verbal. There was nothing, not at all, of clearly physical violence, but definitely verbal abuse more than once because with her, well I had several episodes of… I was mistreated, so to speak. She yelled at me or in front of people when we went out for a drink; so, I raised my voice, she didn’t answer me, she made me look bad in front of people, when we argued about an issue."* Participant 9. Bisexual woman, victim of DV.

The same participant states that at the beginning of the relationship, she did not identify the situations of violence as such and that she tried to justify them; this is a reiterative fact in other interviews with different participants:

"… *However, in the end, I did not realize those things because I kind of normalized them; I thought that well, nothing really happens when she talks to me like that, why she tells me those things, because I know that she truly loves me. I started to normalize that she’s just like that because I’d say it’s just that she’s very aggressive, she gives attitude to everyone, nothing happens. But actually later, of course, I got to stay with the good part of the relationship. Then, in the end later, its good until it explodes, and something suddenly comes to you because you didn’t realize” Participant 9. Bisexual woman, victim of DV.*

*“At first they were just comments, so I thought; I mean, the guy’s still jealous. But, then, little by little he got more… And then later, he also started to be really possessive. Because many times I’d be on my floor and suddenly he’d say to me, open up, since I’m on the floor. So at first, I thought how silly of me, how nice! That he comes to see me, what a nice thing to do. But, no, then I realized what it was about, well, to keep me under control.” Participant 5. Heterosexual woman, victim of DV.*

This shows how difficult it is to identify DV from when it starts by those experiencing it and by those who are close to those who suffer, pigeonholing from the beginning these situations as normal or as couple-things.

*“Many times, they think that they are like fights between the couple, or perhaps, they even do it playing or jokingly. However, that is until you get to know the people in the relationship well. I mean, outside of that relationship, it is also difficult to identify it."* Participant 11. Heterosexual man, DV witness.

*“I thought my relationship was good; well, I don’t know. It was normal, in quotes, with its couple-issues and then, however, that’s actually what caused me anxiety."* Participant 2. Heterosexual woman, victim of DV.

After analyzing the verbal testimonies of DV victims, the difficulty in identifying DV for victims themselves is observed. Victims of DV normalize behaviors such as insults, shouting or disrespect because they consider that by not suffering physical violence they are not suffering DV. And they classify their relationships as good or normal.

### Manifestations of violence

Regarding the different types of violence experienced by the different participants, we found a large number of manifestations of psychological violence, such as attitudes of control, manipulation, emotional blackmail, jealousy or disrespect.

*“The plan at the beginning of everything, always like everywhere else, is really nice, right?But, then they come, those irritations or whatever, foolishness to call it something, that in that moment you don’t realize, but then, deep down, they are actually manipulations that that person does to make you do what they want. What do I know about this person. That is why I did a lot of things being influenced, and I stopped doing a lot of things, to the point of stopping talking to my friends, my lifelong friends."* Participant 2. Heterosexual woman, victim of DV.

“*They crush you daily telling you how horrible you are and how bad you do everything and that you are worth nothing, and in the end, it ends up taking away your desire for everything. You end up feeling like a piece of shit, that you are worth nothing, that you do not deserve to live or even be alive."* Participant 3. Heterosexual woman, victim of DV.

Control was often carried out through social networks, finding a variety of examples.

*“The only thing we knew was that he had my friend's username and password, so he controlled everything he did; on the contrary, she was not like that, that is, she did not have his, but he has hers, so he controlled who she spoke to that way*." Participant 10. Homosexual man, witness of DV.

*“Yes, of course, he controlled me. My time on WhatsApp, I had to have it updated all the time because he had to see when I was online, if maybe I was connecting late or maybe I was connected; he wondered what I was doing and with who I was talking to. Or for example also with Instagram, if I could not connect to WhatsApp, I connect to Instagram and the connection goes out. What? What were you doing, what were you doing up so late, if I’ve gone out, if I’d done such and such? In other words, so paranoid, yes*.” Participant 2. Heterosexual woman, victim of DV.

The same things happens with verbal abuse, encountering a lot of shouting and insults.

"*Yes, once she insulted me; she called me some kind of scumbag because she has a very, very strong character and from time to time it escapes her*". Participant 8. Heterosexual man, victim of DV.

“*Yelling at me and yelling in general. Both. However, basically it was whenever you disagreed a little bit or said something that was not, was not very funny to him."* Participant 3. Heterosexual woman, victim of DV.

We also find examples of social violence, where those who suffer it are deprived of their closest social environment.

*“And I don’t know, I mean, it’s that I couldn’t do anything; it’s just that, I couldn’t even talk to my friends, I couldn’t go out with my friends. My only excuse for seeing them was, you know, a birthday of my friend so-and-so. I said I’m going to go out, and he got angry, saying that he doesn’t go to his friends' birthdays, and I say, fine, so, let’s see, you say, you will not go because you do not want to, but I want to see my friends*". Participant 2. Heterosexual woman, victim of DV.

*“… I would say it’s more blackmail than anything else, emotional blackmail, because she already knows what she means to me and that I gave everything for her. And if I had to leave my friends for two days, well, I would do it to go with her, you know? And she messed with that a lot, with the idea that I was whipped and I loved it. I was super in love, and she played a lot with it… Anything I did that was not with her, anger. It pissed her off. So, because it’s always been very ‘I want everything for me’, I don’t know how to speak of the actual word of being very possessive with me, you know?*” Participant 8. Heterosexual man, victim of DV.

There are fewer manifestations of physical violence found, going more unnoticed for not causing harm to the other person.

*“For example, going somewhere in the car and my partner was driving and starts an argument because many times, he would get angry by himself and start yelling. As much as something even a little bit off, and in the car, hitting the wheel; once at home, also kicking a chair to break it; ripping off his shirt too and tearing it; and yelling, yelling a lot, yelling*". Participant 3. Heterosexual woman, victim of DV.

*“She was very aggressive when she got angry for other reasons, not for anything related to me, or maybe she would punch the wall or things like that to release energy, but I stayed a bit… And yes, it’s true that there was a time when we faced each other, and we went head to head as if we were about to say let’s fight. However, nothing came of it*." Participant 9. bisexual woman, victim of DV.

As has been illustrated with the previous examples, that among the selected population group the most common is psychological violence through acts such as control, shouting or verbal violence, followed by social violence with actions of isolation of the victim. Physical violence remains in the background, without harming the victims physically through its direct use.

### Consequences

Among the consequences suffered and observed from being a victim of DV, we find that anxiety is the most common, manifesting in different ways among the participants and seriously affecting those who suffer it.

*“… I started to breathe, breathe, breathe, and of course, it overwhelmed me; it is like you start to breathe a lot and can’t catch any air. You get overwhelmed, and I don’t know. That was the feeling then, of course; my chest began to hurt and of course, in the area around my heart and such, it hurt a lot, and not only when I had the anxiety attack but also at many other certain times; it happened without doing anything… A couple of attacks happened, and yes, I went to the hospital, and that is it; well, they referred me to a consultation. Maybe it could be a heart problem, even though I was young, but then in the end, it turned out that it was anxiety, and of course, it shocked me because I thought my relationship was good. Well I don’t know. It was normal, in quotes, with its usual partner stuff and then, however, it was that which caused me anxiety.”* Participant 2. Heterosexual woman, victim of DV.

These consequences make those who suffer DV reflect on their relationship and consider leaving it, although many times they do not succeed.

*“… We had a big fight over something silly, and I had an anxiety attack because I kind of got angry, and I said look, and I started to breathe, breathe very hard and say don’t stay, that I had to get out of the car, I was with the car and that night, I said stop… I said, I have to leave because I do not know what is happening to me; I am going to leave, but no, I was not able, I was not able, I did not leave her."* Participant 9. bisexual woman, victim of DV.

Other consequences that we find are depression, stress, insomnia, nightmares, lack of appetite, initiation of smoking and poorer academic performance.

“*I was horrible. I had anxiety. I had a lot of stress. And even, I think depression, because I said, I told my best friend that I’m in a bad way. This guy does not help me; he makes me feel worse. And in fact, I hardly even went to classes. How strange for me because I was alone; I am always one of those who go to class. And, well, those two months, terrible. I think I also need to lose 4 kg, just because of the stress it caused me."* Participant 5. Heterosexual woman, victim of DV.

*“Yes, I did not sleep. Stopped eating; I am one person, also, I love food; I enjoy food a lot, and I love it. I stopped eating and lost 10 or 15 kg… Well, I was missing a lot of class, too.*

*And when I went to class, what I did was sleep, sleep, sleep. I slept a lot, slept a lot because it meant a lot to me, and it was good for me. For me, sleeping was my, my escape route… I could not study; I sat down and could not study. I was very thirsty and had many anxiety attacks.*

*Additionally, I don’t smoke. Do I smoke some at parties? Well, I smoked… I even bought a pack, I would smoke it in one afternoon. I started smoking in that sense*.” Participant 6. Heterosexual woman, victim of DV.

Anxiety was the main consequence of the victims after suffering DV, even requiring medical attention, but it was not the only consequence that has been observed.

### Seeking help

The process of seeking help for people who suffer from DV is complicated, often not seeking help until the relationship ends. When they start the process of seeking help, they usually turn to their closest environment, such as friends and family.

“*I did not tell anyone about all of this, and in fact my parents, when they found out that we had broken off the relationship, because it was also very hard for me to tell them, because he came to my house a lot. Well, they were surprised because they did not know anything. Also, of course, when I told them, well I collapsed, and I started crying. In fact, sometimes when I talk about it, well, it makes me want to cry, not because I think how stupid I was, and then let’s see, but also because it was also something so hard.*

*And, my friends, I also didn’t tell them, not even my best friends, because he was always there, hammering it into me that I don’t need to tell anyone about our discussions. However, now I say that I was stupid for not telling anyone, not even my mother, because who are you going to tell your problems to, but your mother, who is going to advise you the best? I didn’t even talk to her about it."* Participant 2. Heterosexual woman, victim of DV.

Other times, we find the victims supported in activities that help them escape from the reality of the relationship, such as sports.

*“Well, I did play sports. I have always played volleyball, and in fact, I still play. And, I liked it a lot. Sometimes, I could go for four hours to train, but I could only go one day and then I would go for more days, I would also go, and it made me feel alive because I disconnected, I forgot everything, I had a good time*". Participant 2. Heterosexual woman, victim of DV.

It is the family and friends to whom they turn many times who make them see that they are going through a situation of violence and who advise them to leave the relationship, although the affected people do not want to and therefore these tips do not serve them in any case and only help to unburden themselves emotionally.

“*When you’re in it, 800,000 people can tell you to get out of there, let’s go, that you’re in a super deep hole, but until you say that’s it, nothing, it’s impossible. My friends, my family and everyone told me that I was worth something, and even so I, because that is what I told you at the beginning, was afraid of leaving and being worse outside than inside*." Participant 3. Heterosexual woman, victim of DV.

*“I lived in a student residence, and my classmates from the residence hall, of course, saw me coming back, maybe crying home or I would come back and tell them about it, I don’t know what, she has done this and more and I don’t know how much. And, they said "X", wake up, open your eyes. Don’t you see the bad she’s doing?” The truth is that I am going to be honest; it did not help me at that time that they said look, leave it now because they clearly told me you have to leave now, you are not doing well, and of course, that didn’t suit me because I didn’t want to leave her… Well, I didn’t listen; it went in one ear and out the other, so I didn’t pay attention to anything of what my friends were advising me."* Participant 9. bisexual woman, victim of DV.

The process of seeking formal help involves seeking professional psychological help and even psychiatric help. This help often comes through family and friends who have been sought for help.

*“I left thanks to my father, who is a psychiatric worker. I spoke with a psychiatrist, and I was able to leave. I was in several sessions at the beginning, but at first when I left the relationship I felt renewed, then it was like… And then, it was much later when I was worse; but hey, it also helped me a lot to go to even a few sessions with the psychiatrist. He gave me some advice that I still carry mentally to balance myself*". Participant 6. Heterosexual woman, victim of DV.

"*It had several serious consequences until going to a specialist, and with the help of the specialist and the family, because it is like the relationship was cut off for good*". Participant 10. Homosexual man, witness of DV.

In turn, there are those who point out the reasons for not seeking professional help, including a lack of financial resources and a fear of facing the situation.

*“The truth is that I would have liked to go, but I do not have any money. If you had said that they would have given me an appointment in the public health system, perhaps maybe. In fact, today I still want to go, but I do not have the money to pay for it. Maybe I can pay for one session, but I cannot pay more*." Participant 9. bisexual woman, victim of DV.

*“No, but I did think about it many times and then didn’t go through with it. I think that by not worrying my parents or by making myself think that the situation didn’t deserve more importance than it actually did, that is, it truly deserved more importance than what I gave it, but I didn’t want to*". Participant 8. Heterosexual man, victim of DV.

The search for help by victims of DV is a complicated process since, as has been stated previously, they first need to recognize that they are suffering violence. Initially this search involves the closest supports such as family and friends. Normally it is this informal support who usually advises and guides the victim towards formal help such as medical resources and psychological treatment when necessary.

### Proposing help

When asked during the interviews what victims and people close to DV would do as future health professionals to help solve or lessen this problem, they responded around two fundamental axes:

- a) Health education in violence, highlighting that violence is not only physical:

“*I think it should be talked about more, especially in these cases of social violence. In terms of isolation, *etc*., or psychological violence, because, of course, we know that as soon as they put their hand on you, you have to report it. However, many times, if you are being insulted or they isolate you socially, we do not resort to that step and therefore we normalize it. So, I think we should talk, for example, the talk that I remember being given at school: for example, a Civil Guard will always talk to me about physical violence, but I have rarely heard about psychological violence. So, I think that much more should be said about the types of violence in general and that they are all equally important, though exercised in different ways, but that they should be denounced anyway*". Participant 4. Heterosexual woman, victim of DV.

*“One of the things that made me realize that I had suffered verbal abuse was one day in class, a talk about things that can be considered verbal abuse… And I began to count them and said I have experienced eight out of ten. No. Me no. I did not realize it then… I think they have to keep giving a lot of talks, especially also personal experiences, because I think there are few… With talks, with support, with reaching out, with trust, especially in parents, who I think are also pillars that can help a lot.*

*We, as health workers, I think that we should give talks about everything and share close testimonies, provide private appointments if they need to talk about it, and also create intimate environments in that sense "* Participant 5. Heterosexual woman, victim of DV.

- b) Effective communication between the couple, with professional help if necessary, and with professionals to aid in the early detection of the problem:

"*Before doing this kind of thing, the first thing to do would be to talk and try to reach a consensus, an agreement or let’s say a conversation to try to solve the problem; but, I think that is why I have seen it not frequently done…Let’s see, I understand that first, you have to reflect; then, I think it is okay to ask for opinions on how it looks from the outside, as with the version that you are giving to see. What do people think? And if you already see that it’s a problem that you cannot solve with your partner and already, well, you have two options: either end it or you go to a psychologist for advice, maybe a little better, since they have more knowledge about it. When you see that a person is wrong, try to talk to them and try to listen to them… Also, above all, see what the problem is then. Once you know what the problem is, I would already offer, well what I would do is go to a professional who can advise you on this so that you can handle it as well as possible because it is true that sometimes these situations escape you and you can have them in front of you and not realize it."* Participant 7. heterosexual man, witness of DV.

*“The first thing I would say is how to get more information about it because maybe at first, they don’t tell you. But maybe these signs indicate that some type of abuse is taking place and then investigate a little, not without being too intrusive within the relationship. However, by gathering enough information, you should be able to act on it*." Participant 11. Heterosexual man, DV witness.

### Description of Dv

Among the testimonies obtained, it is worth highlighting the harshness with which DV victims describe and define what it is like for them to have suffered DV.

*“For someone to take away all the self-love you have, to lose all the self-love that a person can have, the feeling that you are trapped, that you are trapped somewhere, but you cannot get out of there, and it’s in that place that we are always trying to get out. But you feel like if you get out of there, you will be even worse*.” Participant 3. Heterosexual woman, victim of DV.

*“Well, I would describe it with one word. It’s hell. Because, at the end of the day, everything, that is, all the comments, which are the little ones the other person says or all the things that the other person does who supposedly loves you makes you feel bad, they make you beat your brains out, and I think that’s what it’s like to fight with your own demons. "* Participant 5. Heterosexual woman, victim of DV.

“*I would define abuse as a state of helplessness and self-cancelation. That with acts that you don’t realize and that are canceling you little by little, little by little. And, they make you… They make you forget who you are completely."* Participant 6. Heterosexual woman, victim of DV.

People who had not been victims of DV tried to give a definition closer to the theory:

“I *would say that it is any act, be it physical or psychological, that can affect the health of the other person. And, in this case, as we are talking about intimate partners, then within the relationship*.” Participant 11. Heterosexual man, DV witness.

### Violence and gender

At the end of the interview, the participants were asked about the influence of gender on suffering violence, obtaining fairly uniform responses, stating that although gender is traditionally an influencing factor and that it is usually men who exert violence, men can also be victims of DV.

"*There is a lot of it, and very few times that violence is toward men; this is not very common to hear that because what is always seen the most is physical violence… Most of the ones who carry out physical violence are men or when it comes out in something on the news or on some show or something. However, really what people don’t think about though is psychological violence, to which many men are subjected by women… Well, obviously, in the end what also happens is that society often gets the idea that If you complain, what are you going to complain about?"* Participant 1. Heterosexual woman, DV witness.

*“Let’s see, let’s see, I don’t know. Normally or what we understand is that men tend to do violence against women. However, there are also many times when it’s the other way around, but of course, it is one of those cases that are less talked about. But for certain, there is also a lot, but people may not speak about it because of the feeling of, I don’t know, perhaps shame or because how am I going to tell anyone if it was a woman who’d done it to me? But, of course, I mean, just as there are men like that, there are also women like that."* Participant 2. Heterosexual woman, victim of DV.

“I *would say no, the truth is, although by statistics and percentage and everything, it is always the man, the one who always exercises control over women, always the same. I think that in the end it depends, well, much more on education than simply the gender of the person. Though, it’s true that currently there is much more violence by men than women, obviously, but it does not mean that men are violent because they are men or that women are another way because they are also, you know, independent*". Participant 8. Heterosexual man, victim of DV.

Throughout the results, the DV situations of nursing students have been exposed with real verbal testimonies.

Finding testimonies about the beginning of violence, its normalization and difficulty in recognizing it, the types of violence and its manifestations, among which it is worth highlighting situations of control. The consequences that having suffered DV have had for these students and how they have sought help when it has been necessary. Likewise, it has been exposed how they describe what it is like to suffer DV, and as nursing students, how they believe that this problem should be intervened and finally how they relate gender to DV victimization.

## Discussion

This study tries to understand the experiences of university students with regard to DV, observing a serious problem about the lack of recognition of DV situations and the normalization of violent situations among university students, having consequences such as anxiety and seeking help mainly in the immediate environment at the end of the relationship. Several studies investigate the minimization of DV [35–37], and the results indicate that the degree to which victims accept the situation makes them incapable of describing their relationship as abusive and leads to them justifying the violent behaviors. According to a study carried out by Fernandez-Antelo et al., the more frequent the abuse, the greater is the acceptance of violent behaviors and the greater is the risk of being a victim of violence again [36]; the study also suggests that men are more accepting of aggressive behaviors than are women, possibly because they see themselves as potential perpetrators of violence or because they still prefer to stay with their partner. Along these lines, another study carried out with nursing and health sciences students found that men showed more sexist attitudes and ways of justifying violence and, in turn, higher rates of DV [38].

As in our study, when asking about violence through social networks, we found it difficult to identify behaviors of harassment, control and abuse by partners toward DV victims [39]. Sanchéz-Hérnandez et al. indicated that university students are able to recognize controlling behaviors through e-media in the role as observers of other couples but that they do not recognize that they suffer or exercise this type of behavior within their own relationships. We conclude that this type of behavior is accepted and normalized in this population group. This agrees with the results obtained by other researchers, such as Kirby CM, et al. (2022), who also adds that beliefs and myths surrounding psychological violence also contribute to its normalization or minimization of the problem [40].

Along the same lines is the study carried out by Temple et al. [35], who investigate the relationship among psychological abuse, the normalization of violence and mental health problems such as depression and anxiety, obtaining significant relationships among all of them. In this study, anxiety was the main consequence described by the victims of DV interviewed, and they encountered difficulties to overcome it. Other studies on the relationship of DV with depression and anxiety [13, 41, 42] found anxiety to be a consequence of suffering DV and poorer mental health states, consistent with our results.

Another of the key points regarding DV is the process of seeking help. From the data extracted from the interviews, victims usually go to their immediate environment in search of informal help; however, this process occurs when the relationship has already ended. Fernet et al. [22] study this process, clarifying that it begins with defining the problem by those who suffer it, followed by the decision to seek help and finally choosing a source of support. Informal support networks offer emotional help, protection when the situation requires it, and support with practical skills and material resources. In turn, the author identifies the reasons for asking for help from peers: seeking advice to solve problems, expressing emotions and feelings, obtaining an outside point of view, being listened to and comforted, and validating a correct interpretation of the situation.

Regarding the choice of the source of support, within the process of seeking help, as in our study, Madkour et al. [29] found that in African American students, adolescents who suffer from DV turn first to seek help from their friends, followed by family and, last, other adults or professionals. It also indicates that a high percentage does not believe that it is necessary to report these types of issues.

Other studies investigate peer support in college students who suffer DV. According to Cusano et al. [28], peer support is influenced by the recognition and identification of toxic and abusive behaviors, determination of the risk for their peers and the anticipation of the possible consequences after the intervention. This study in turn identifies the main barriers to intervening once these situations are detected: lack of training on the subject, existence of abusive behaviors not legally punished, such as psychological or emotional abuse, and lack of recognition of the situation by the victim.

According to a review carried out by Debnam and Mauer [26], informal help will be provided by those with the greatest sense of responsibility and trust for intervening or who are directly involved and know the people involved.

In the interviews, several participants claimed to have received advice on leaving the relationship; on this subject, we found a qualitative study carried out by Ermer et al. [43] on forgiving or leaving a relationship with DV, finding that the type and severity of the aggression, the context and the frequency with which the violent actions occur do matter.

Regarding the influence of gender on DV in the quantitative studies consulted, it is clear that both genders suffer DV equally without significant differences [11–14]; however, this does not explain why the belief that men are always or usually the aggressors and women the victims still persists. According to a mixed study carried out by Medina-Maldonado et al. [44], differences are found between qualitative and quantitative data, as in our results, because the male gender is identified as the perpetrator and the female as the victim of DV. Another qualitative study conducted by Taylor et al. with focus groups [24] found that men tend to understand violence in terms of action and physical consequences, placing themselves, in the social point of view, as perpetrators of abuse, while women understand violence in emotional terms, focusing violence toward a social and family plane and placing themselves, in the social point of view, as the victims of violence.

Regarding the role of gender in DV, an interpretive meta-synthesis of the qualitative literature carried out by Storer et al. [25] establishes this as a social phenomenon and linked to the context. The results obtained indicate that gender affects the impact of violence and that gender-specific actions are linked to violence, such as the fact that a male using physical violence is seen as unacceptable while, on the contrary, a female using violence it is more permissible.

The nursing students who participated in this study proposed ways to aid in reducing DV, highlighting greater education in violence and strategies for effective communication. The DV prevention programs found were *Dating Matters* [45, 46], *Fourth R* [47]*, Safe Dates* [48] and *Shifting Boundaries* [49] and target adolescents in schools and institutes and have been carried out mainly in the United States, seeking ways to create healthy relationships between adolescents through knowledge and skills. In Spain, the intervention carried out by Sánchez-Jiménez et al. [50, 51], i.e., *Dat-e Adolescence*, evaluated the factors associated with DV, such as self-esteem, myths about romantic love and the regulation of anger, but did not investigate the prevalence of DV after the intervention. So, it is necessary to create new prevention programs at the university level.

## Conclusions

The experiences of university students about DV are mainly painful experiences, with serious consequences for those involved that affect the health of the victims, needing help from their close environment and professional help to overcome the problems generated by their partners with aggressive behaviors, control, manipulation, harassment, social isolation, etc.

Therefore, it is necessary to raise awareness surrounding the problem of DV; the lack of awareness is evident in the absence of recognizing violent situations by victims of DV and the justification of nonphysical violence behaviors as "normal" within relationships.

For this reason, it is also necessary to expand general knowledge about violence, emphasizing for everyone, and specifically young people, that violence is not only physical and that other types of violence also have serious consequences for those who suffer it. For this reason, more training programs should be implemented, and relationships with consequences as serious as suffering anxiety should be discussed, providing examples of people close to whom it may be happening.

It is important to have a close and familiar environment in which to seek help when needed and important, in turn, to know how to react to the situation and provide solutions or help that a victim is willing to accept. The importance of formal help, such as psychological help, should be emphasized.

Professional health care workers have to improve interventions for violence, improve health education pertaining to these issues, carry out prevention campaigns and undergo training to be able to detect situations of violence at the early stages. In addition, improvements in communication tools can help students develop active communication skills that they can use in relationships with peers and with intimate partners.

Importantly, the actions that are carried out to help reduce this problem should not be biased due to gender and sexual orientation because these are not influencing factors for suffering and perpetrating DV.

DV is a serious problem that should not be underestimated, seriously affecting those who suffer from it, as evidenced by victims describing the problem and their experience using devastating words.

### Implications

The results obtained through the interviews reveal the seriousness of DV for people who are victims of this type of intimate partner violence, seriously affecting the lives of those who are involved and needing both formal and informal help to face the problem and overcome it.

There is a need to create programs against DV to prevent and be able to act against it and to provide key points to future health professionals and victims of DV on the correct way to act when we are faced with this type of situation. These programs need to be aimed at the target population and not discriminate based on gender or sexual orientation because it is clear that being a victim or perpetrator of violence does not depend on these factors.

It is necessary to continue researching DV and its associated factors, consequences, and protective factors to better understand this phenomenon, in order to help solve the problem in the most appropriate way.

### Limitations

This study has some limitations that should be addressed in future studies. The main focus of the study the experiences of the victims around DV; however, not all participants in the interviews were victims of DV, although those who had not experienced DV, they had experienced it with friends close to them and/or had been perpetrators of DV. Although the sample size is small, there was data saturation in all starting categories. Since the interviews are semi-structured and work with self-reported data, the individual veracity of the information provided cannot be checked. In addition, since the interviews are conducted online, the physical location of the interviewer could not be controlled and the privacy could not be guaranteed, although the researchers guaranteed privacy in the recording of the interview. We think that online interviews can be useful for these types of people who may feel controlled by their partner, intimidated by fear or embarrassment to go to a physical face-to-face space with the interviewer. Additionally, researchers may introduce bias when conducting qualitative research.

Among the strengths of this study are having a qualitative sample representative of the population of nursing students who suffer DV, having people of both genders and integrating sexual diversity into this study. Furthermore, by conducting the interviews online and recording them, the risks of loss of information and transcription errors were reduced.

### Supplementary Information

**Additional file 1. **Interview guide.

## Data Availability

The datasets generated and/or analysed during the current study are not publicly available due to maintain the individual privacy of the participants but are available from the corresponding author on reasonable request.

## Abbreviations

DV: Dating violence
WHO: World Health Organization
