# Supplementary material for: Experiences of nursing students who are victims of dating violence: a qualitative study
Source: BMC Nurs. 2024 Jan 9;23:28. doi: 10.1186/s12912-023-01688-w (PMC10775457; doi:10.1186/s12912-023-01688-w)
Supplement: Supplementary file 1 — Additional file 1. Interview guide. [file 12912_2023_1688_MOESM1_ESM.docx]

The interview began by asking participants to talk about their experiences with dating violence. If they did not know where to start or the information provided was scarce, the questions set out below continued to be asked.

- What is your opinion on dating violence? Do you have any personal or known experiences on the topic?
- For you, what is dating violence?
- What aspects of your relationship or the one you've spoken about do you identify as violence? What types of violent situations and/or aggressions have you identified?
- Was there any violence through social media in the previous case? (e.g., monitoring your social media, harassing you through it, deciding what content you could post...)
- What signs of violence were the first ones you identified in this relationship? Why?
- Have you tried or do you know if this person tried to end the relationship? What problems have prevented you from leaving the relationship?
- Was help sought while it was happening? Was the issue discussed with someone? Was the case reported?
- When you've talked about this with someone, how has it helped you? In case you've experienced or might experience violence in the future, how has talking to someone helped or would help you? Whom have you turned to or would you turn to for talking?
- How did you face/would you face the situation in these situations you've mentioned?
- Did it affect you personally in any way? Have these situations changed your daily life? Have you incorporated new habits into your life to cope with them?
- Have you managed to solve the problem in any unexpected ways?
- Do you believe that this type of situation is tolerated and socially accepted? Why?
- Do you think gender influences the likelihood of being a victim of dating violence in any way?
- What do you believe healthcare professionals can do for people who have experienced or are currently experiencing this type of situation?
- Do you think there's anything that hasn't been covered in the interview that you would like to add?
